# Moral Distress in the Neonatal Intensive Care Unit: What Is It, Why It Happens, and How We Can Address It

**DOI:** 10.3389/fped.2020.00581

**Published:** 2020-09-10

**Authors:** Manisha Mills, DonnaMaria E. Cortezzo

**Affiliations:** ^1^Division of Neonatal and Pulmonary Biology, Cincinnati Children's Hospital Medical Center, Cincinnati, OH, United States; ^2^Division of Pain and Palliative Medicine, Cincinnati Children's Hospital Medical Center, Cincinnati, OH, United States; ^3^Department of Pediatrics, University of Cincinnati College of Medicine, Cincinnati, OH, United States; ^4^Department of Anesthesiology, University of Cincinnati College of Medicine, Cincinnati, OH, United States

**Keywords:** moral distress, decision-making, neonatal intensive care, ethical confrontation, periviability, end-of-life care, medical futility

## Abstract

Moral distress is prevalent in the neonatal intensive care unit (NICU), where decisions regarding end-of-life care, periviable resuscitation, and medical futility are common. Due to its origins in the nursing literature, moral distress has primarily been reported among bedside nurses in relation to the hierarchy of the medical team. However, it is increasingly recognized that moral distress may exist in different forms than initially described and that healthcare professions outside of nursing experience it. Advances in medical technology have allowed the smallest, sickest neonates to survive. The treatment for critically ill infants is no longer simply limited by the capability of medical technology but also by moral and ethical boundaries of what is right for a given child and family. Shared decision-making and the zone of parental discretion can inform and challenge the medical team to balance the complexities of patient autonomy against harm and suffering. Limited ability to prognosticate and uncertainty in outcomes add to the challenges faced with ethical dilemmas. While this does not necessarily equate to moral distress, subjective views of quality of life and personal values in these situations can lead to moral distress if the plans of care and the validity of each path are not fully explored. Differences in opinions and approaches between members of the medical team can strain relationships and affect each individual differently. It is unclear how the various types of moral distress uniquely impact each profession and their role in the distinctively challenging decisions made in the NICU environment. The purpose of this review is to describe moral distress and the situations that give rise to it in the NICU, ways in which various members of the medical team experience it, how it impacts care delivery, and approaches to address it.

## Introduction

American philosopher Martha Nussbaum writes “in all situations of choice, we face a question that I call ‘the obvious question': what shall we do? But sometimes we also face, or should face, a different question, which I call ‘the tragic question:' is any of the alternatives open to us free from serious moral wrongdoing?”(1) In Nussbaum's “tragic question” lies the crux of moral distress. This concept in relation to the field of medicine was first described and defined by Jameton in 1984 as “the psychological distress of being in a situation in which one is constrained from acting on what one knows to be right”(2). Though its first applications were primarily limited to nursing practice, the concept has since been broadened to include other groups of healthcare professionals.

The concept of constraint is central to the essence of the original definition moral distress and is a predominant theme in nursing literature. Moral dilemma or conflict, on the other hand, is more often reported as being experienced by physicians (3). In circumstances where there is moral conflict, values systems or duties relating to multiple treatment options are incompatible with one another and lead to psychological distress (4). The provider feels he must act in a way or provide care that is contrary to what he believes is the appropriate care plan (5). This discrepancy inherently challenges one's ethical principles. As a result, the term ethical confrontation has also been used to describe the associated distress (6). In an effort to provide the most inclusive review possible, moral constraint, moral conflict, and ethical confrontation will be considered in the discussion of moral distress.

While it is necessary to expand the definition of moral distress beyond the idea of moral constraint as providers are rarely faced with one definitive action on which to make a moral or ethical judgement, there must be a clear distinction between separate albeit related concepts of uncertainty, ethical dilemmas, and moral distress. In the neonatal intensive care unit (NICU), there is seldom absolute assurance in the outcome for a baby with the choices or treatment options available. There is always an element of uncertainty in outcomes that can lend itself to varying degrees of distress when discussing treatment options and decision-making with families. In addition, the NICU is wrought with ethically ambiguous clinical circumstances and complex decisions for a vulnerable population. This innately leads to feelings of internal discord, powerlessness, and uncertainty in physicians, nurses, and other healthcare professionals. This type of uncertainty, while undoubtedly causing stress and discomfort, should not be confused with moral distress. While there are times when these situations lead to distress if values are challenged, the presence of uncertainty or ethical dilemmas do not automatically equate to moral distress.

Frequently, though, decisions regarding end-of-life care and life-sustaining measures provoke moral distress, requiring a closer look at how this ultimately influences the care that is provided. Providers may feel constrained by the uncertainty in these decisions and having to counsel parents with limited information about the possible outcomes. Regardless of the etiology, moral distress can impact patient care and provider longevity (5, 7–9). If addressed, though, providers can minimize the negative affects while supporting each other and fostering personal growth (10, 11). The purpose of this review is to describe the role of moral distress in the NICU, reasons it occurs, how it presents unique challenges to different healthcare professions, and how providers can address it.

## The Scope of the Problem

While it is accepted that moral distress is ubiquitous for those who work in healthcare, the true prevalence in the NICU is not well-established (12). Some historical challenges in assessing the prevalence include a focus in the literature on parental moral distress and adult patients, multiple definitions of moral distress, and an emphasis on the nursing profession (13, 14). While moral distress can affect any member of the healthcare team, it is most prevalent in those who provide direct care, such as physicians and nurses. This is likely due to the close relationships formed with patients and their families and the sense of responsibility in providing care (15). As such, it is increasingly recognized that moral distress can affect any member of the medical team regardless of profession.

For nurses, common causes of moral distress can be patient-focused or nursing-focused (16). Patient factors largely focus on quality of life, advocacy for the patient, and communication challenges with the families and care team. Nursing factors may include not having a voice within the care team, unclear roles, personal or team conflict, and feeling their integrity is being compromised. Historically, it was thought that the factors leading to moral distress were rooted in paternalistic approaches to medicine where nurses were instructed by physicians to provide care they did not always feel was appropriate (5).

With newer multidisciplinary care models, a recent focus on understanding healthcare provider moral distress has given more insight to the scope of this issue. In a recent qualitative analysis of attitudes around moral distress of NICU physicians and nurses, it was reported that up to 72% of providers experience moral distress at least once a month (10). Other studies show similar findings with up to 58% of nurses and physicians reporting specific work related moral distress (8). While it presents differently for each profession, all members of the medical team are vulnerable to this phenomenon.

Despite the prevalence, there is significant variability around the degree and frequency at which providers experience moral distress or ethical confrontation. This is likely due in part to the characteristics of the individuals included in studies. One study evaluating the impact of experience and knowledge of providers on the frequency of ethical confrontation reported that 35% of experienced NICU nurses and 19% of pediatric or obstetric residents experienced this challenge frequently at work. Overall, in the presence of ethical confrontation, greater experience and a higher level of understanding were associated with lower rates of moral distress. Perhaps with experience providers are able to process and cope with ethically challenging situations in a manner that they do not feel moral constraint. On the other hand, increased knowledge about particular medical situations can open the possibility of experiencing moral distress. Individuals at medical centers that reported lower rates of moral distress around periviable resuscitation were less knowledgeable about outcomes for extremely preterm infants and were less likely to offer resuscitation at the lowest gestational ages. Those with more experience in the consequences of extreme prematurity, especially nursing staff, tend to overestimate poor outcomes and have higher rates of moral distress around offering resuscitation. Overall, the culture of a given center had the greatest impact on levels of moral distress, with the lowest frequencies of moral distress reported at centers that were the most homogenous with respect to language spoken and religious affiliation (6). This is in contrast to the longstanding notion that moral distress primarily originates from medical team hierarchy and suggests that heterogeneous values and background may contribute more to moral distress than was originally thought. Results of this and similar studies varied considerably with respect to the frequency and intensity of moral distress experienced by varying members of the medical team (6, 8, 12, 17).

Interestingly despite the challenges associated with moral distress, up to 76% of healthcare professionals feel it is a necessary part of caring for critically ill neonates (10). They view this feeling as a byproduct of being a caring, compassionate provider who is invested in the well-being of their patient and providing the best possible care. It is also viewed as a sign of innovation and progress in patient care (11). The internal struggle created by a difficult situation can, at times, be necessary. It challenges providers to acknowledge their own distress and biases in caring for patients and talking with families. This is important because the information communicated to families and the manner in which it is communicated can impact decisions made and ultimately lead to transference of distress (5). Simply because the medical team and/or parents agree upon a specific treatment course does not guarantee the accuracy or benefit of the treatment. Without the challenge of differing opinions, the ethical, moral, and medical appropriateness of such a decision goes unscrutinized (11). Discussions that emerge from moral distress promote exploration of multiple courses of action and encourage the most informed decision possible.

While providers feel that moral distress is an innate part of caring for NICU patients, it is accompanied by some degree of burden. Physician trainees in the NICU reported, through longitudinal narrative writing, that they experienced conflict in multiple situations that caused them distress and led them to question their own morals (18). The unique position of trainees in the medical team hierarchy places them at risk for constraint distress. They may feel obligated to provide care they do not agree with and are unable to voice these disagreements due to lack of confidence, fear of unfavorable evaluation, or concern for retaliation. As a result, they may feel numb and cope through detachment and desensitization, which can lead to compassion fatigue and burnout over time (18, 19). The emotional toll, which transcends beyond trainees, can lead to disengagement and ultimately effect patient care with avoidance behaviors amongst staff, increase length of patients' hospital stays, adverse patient outcomes, and increased pain levels in patients (12, 20). In addition, moral distress can contribute to increased burnout, decreased job retention by the healthcare provider, decreased staff retention by institutions, career transitions, threatened moral integrity, and a sense of failure to perform a professional and moral duty (5, 7–9). In order to provide optimal patient care and a safe environment for providers to thrive, when moral distress is prevalent providers are faced with a balancing act of minimizing the negative impact and leveraging the potential for progress (9).

## Why is Moral Distress so Prevalent in the Nicu?

Many factors can contribute to moral distress. Intensive care settings, staffing shortages, the need for timeliness/efficiency, and situations involving perceived futile care or different perspectives on end-of-life decisions place individuals at higher risk for moral distress (12). Structural aspects of the NICU, including lack of a consistent care team, poor communication, and understaffing lead to moral distress due to feelings of providing substandard care (21). Beyond the structure itself, variation in providers' own views and values within an institution can lead to reports of greater moral distress (6). Perhaps there are factors innate to the providers themselves and the culture of the institution that play a role in the presence of moral distress. When providers on the same care team have different values and moral compasses, there is a higher likelihood of some team members feeling moral constraint and subsequently moral distress based on the care provided.

The vulnerability of the NICU population, combined with the need for decision-making by proxy further compounds this risk. In the face of advancing medical knowledge and increasing reliance on technology and life sustaining measures, parents and healthcare providers struggle to find balance and determine what is truly in the best interest of the child (22). The frequency of end-of-life situations and the complex nature of NICU care lends itself to moral distress. One study found that a higher number of deaths and issues surrounding end-of-life care and resuscitations are associated with the highest levels of moral distress (8, 17). Interactions between team members and with parents and family can ease or amplify these feelings (17). Similarly, the degree to which medical team members agree with one another and with the parents' decisions can influence perceptions of constraint or control. It is far more common for healthcare providers to feel they are “doing too much” rather than too little. While this can be seen as an ethical dilemma, the resulting concern for inflicting undue harm and suffering results in a greater degree of moral distress (23). Interestingly, while physicians were more likely to disagree with the level of care a patient was receiving than nurses were, their reported levels of moral distress were lower than those of nurses. This may relate to the notion that increased education and experience with these challenging clinical scenarios allows physicians to better delineate ethical questions from moral distress. It also may relate to the fact that nurses are the individuals actively carry out the care plan.

While many factors contribute to how decisions are made for a vulnerable patient who cannot convey their own wishes, as with a neonate, there are external constraints beyond provider judgement that can impact and alter the care plan. Some factors that influence decisions include limitations put forth by laws and regulations, institutional policy, parental wishes and views of quality of life, hierarchy of the healthcare system, or ethics committee rulings. If these factors are in opposition to provider beliefs, it can lead to significant moral distress (25). Similarly, if there is a perception that medical interventions are causing unnecessary suffering, it can lead to feelings of helplessness (26).

Just as important to consider are factors internal and innate to the provider. If there is uncertainty or doubt about the diagnosis, prognosis, or most effective treatment course, it can lead to feelings of powerlessness, fear, lack of knowledge, or hesitation to advocate for their patient (8). These internal constraints are commonly described in the literature but less frequently recognized as sources of moral distress (9, 13, 27, 28). While some have advocated to expand the definition of moral distress to encompass situations beyond where there is moral constraint, these circumstances of uncertainty, even with a broader definition of moral distress are more appropriately described as moral sensitivity or ambiguity rather than moral distress (3, 4).

While moral distress can arise from any complex situation, most common scenarios that give rise to moral distress in the NICU involve end-of-life care, medical utility and futility, periviability, and disagreements about care plans (24). Each of these challenging clinical scenarios, which are described in greater detail below, involve medically complex decision-making.

### End-Of-Life-Care and Palliative Care

There are over 15,000 neonatal deaths each year in the United States and the majority of these occur in the NICU (29). As a result, end-of-life care is routinely tasked to providers who take care of babies in an intensive care setting. Transitions to end-of-life care are never easy and are fraught with grief and uncertainties at a time of heightened emotions for all involved. More than 80% of the time, deaths in the NICU are preceded by a decision to limit, withdraw, or withhold life-sustaining treatments (30). This requires difficult conversations about the prognosis and multiple treatment options and approaches to care available. Once a decision to redirect care is made or it is evident that a patient is dying despite invasive medical interventions, subsequent care can greatly influence parental coping and medical team attitudes surrounding the death.

How providers define or personally view aggressive care, redirection of care, and the reasons for pursuing these care paths can contribute to feelings of moral distress. Singh et al. claim that when an actively dying patient is extubated so the mother may hold the infant as he or she passes, it is ethically different than compassionate withdrawal of care in the setting of a severe neurologic insult with likely poor long-term outcomes. The study also noted that timing of death did not significantly differ for infants who received full support vs. those for whom care was withdrawn (31). Both resulted in the infant's passing, but perhaps, the authors suggest, the former situation offers a clearer assessment of futility and allows moral judgement of providers to align with the actions taken.

The timing of when options such comfort measures, redirection of care, or pursuing invasive medical interventions are discussed is important as it can also impact feelings of moral distress. It may feel different to parents or healthcare providers to withhold care and refrain from initiating a particular care path than to withdraw or redirect care after invasive interventions are initiated. With increasing medical capabilities to care for the tiniest, sickest infants, trials of therapy are not uncommon. Parents may need time to process the information and prognosis, especially with unanticipated events or diagnoses and providers must balance the need to minimize suffering to the patient and utilize resources appropriately. Parents and providers utilize a shared decision-making approach to determine the boundaries of care plans, based not only on prognostic factors, but parental values and goals of care (32). Concern for prolonging suffering while awaiting parental decisions may intensify the medical team's feelings of failure to prevent undue suffering or harm to the patient. Likewise, due to provider discomfort with having these difficult conversations or their own perceptions about the appropriateness of such care plans, discussions often occur late in the hospital course. This can lead to distress and constraint of other providers or the families (32). Moral distress may result from the choice to provide invasive medical interventions, withhold therapies, or withdraw invasive medical interventions especially in circumstances in which there is little data to drive the decision that is in opposition to what the provider believes to be morally right.

Consider a former premature child corrected past term with bronchopulmonary dysplasia who thrives on continuous positive airway pressure and awaits parental decision regarding a tracheostomy to aid in development and facilitate transition to home. Despite declining redirection of care when the baby was critically ill, parents have consistently expressed the desire for an acceptable quality of life over quantity of life. In their eyes, dependence on any medical technology long-term is not an acceptable quality of life. They are increasingly distressed and often in disagreement with the medical team over the amount of invasive care the child is receiving. They express they would not want their child to be dependent on a tracheostomy or gastrointestinal tube feeds for an extended period of time. Ultimately, they elect to redirect care and remove the continuous positive airway pressure mask. The baby dies over the course of a several days. Some of the medical team expresses anguish over withdrawing non-invasive support on a baby with a favorable neurodevelopmental prognosis. They are faced with the constraint of parental desires and request to redirect care despite their own beliefs and views. In the zone of parental discretion, one may choose to honor such a request with appropriate counseling and risk assessment. While there is a level of moral judgement that this treatment course exposes the child to risk and ultimately death, the decision of the parents does not constitute medical neglect as there are also significant risks and burdens associated with the alternate care paths. A child with a tracheostomy who is ventilator dependent requires around-the-clock care and vigilance. They may have significant medical complications including death related to the tracheostomy and long-term ventilator support. In this challenging scenario, the involvement of palliative care and ethics is important. Through their involvement, the care team can better understand the complex factors that went in to the difficult decision made by the parents. This also allows for an avenue for staff to express their concerns, better understand the ethical questions at hand, and process their personal views in relation to the case.

### Medical Utility and Futility

Consideration of medical futility when pursuing life-sustaining measures is important but difficult to apply to a clinical setting. Given the present degree of uncertainty in neonatal outcomes and continual advances in therapies and medical technology available, it is challenging to determine if interventions being provided have a reasonable likelihood of success and what defines success for any particular baby/family. Futility can be defined as a treatment that fails to provide discernable benefit (33). It innately requires delineation of goals as it is a term relative to the desired outcome. This is a challenging concept as many interventions provide some benefit and invasive interventions may allow an individual to live for a period of time. Due to the increasing use of technology and medical innovations, providers may feel that care is at times overly aggressive, without clear or definitive benefit. This can lead to distress and concern that they are causing undue suffering to their patient. It is reported that over half of attending neonatologists and over one-third of nurses have provided care they view as “overly burdensome.” Similarly, 80% of physicians and 69% of nurses felt they had saved a child who “should not be saved,” despite personal and moral objections to doing so (34). The decision of whether or not to continue aggressive care in the setting of a poor prognosis can be morally distressing. In particular, neurodevelopmental outcomes and the impact on long-term perceived quality of life is of great concern when weighing benefit against risk. Simply enabling survival is no longer the goal. A delicate balance between preserved quality of life, incorporating family goals, and minimizing suffering complicates the decision of the most appropriate treatment plan.

Quality of life is inherently personal as what is considered acceptable for one person may not be for another. While physicians overwhelmingly support parental involvement in decisions regarding end-of-life and palliative care, there is a tendency to anchor in the statistical majority with respect to outcomes (35). This can greatly influence how healthcare providers counsel a family and the degree of moral distress following a parental decision that does not align with their own views. In these situations, providers are asking themselves if a life with profound disabilities is a life worth living and if providing life-sustaining measures is worth the cost (36). While weighing these costs, they view what that life could potentially look like. Physicians tend to place a much higher value on cognitive function and independence than families do (37). This is likely, in part, due to the limited context and surroundings in which they interact with children with severe impairments. It can cause moral distress when a provider is challenged to accept different values and provide balanced counseling and at times provide interventions with goals in opposition to their personal values.

Regardless of the underlying diagnosis, when the medical team is asked to provide care that will extend a child's life, they often do so with the best interest of the baby in mind. However, when this is in the context of a child who will have a profound cognitive disability, the best interest standard often comes in to question (38). This is largely based on weighing the burdens and benefits of various treatment pathways for the child in the context of their family. While providers may have personal views, they cannot presume to know if the child would prefer death to a life with profound impairments especially when the child would have no other comparison (39). Often times the treatments available will extend the child's life and potentially facilitate leaving a hospital setting. The interventions themselves do not place the child at risk for imminent, preventable harm. As a result, it is difficult for providers to use ethical principles of best interest and non-maleficence as justification for advocating against such interventions. While there will likely be burdens for the child and family with the pursuit of invasive interventions, some feel these burdens are miniscule in comparison to the life the child is able to have and the potential relationship the baby is able to develop with their family and surroundings (40). With this notion, some advocate for providers to focus on the moral value of that relationship and the capacity for a caring relationship as opposed to neurodevelopmental outcomes when determining if invasive interventions are appropriate. The relational potential for which some advocate is morally meaningful regardless of others ability to see the reciprocity of the relationship (40). It is only with the help of the providers that these potentials can be realized. By acknowledging the significance of this relationship, providers can support their patients' families and potentially feel more comfortable with the interventions they are being asked to provide.

Often, providers find themselves in the zone of parental discretion when the risk of an option does not clearly outweigh the benefit. In such circumstances, parents are assumed to have their child's best interest at heart and know what is best for their family. As a result they are encouraged to be the decision-makers for their child unless the decision will cause express harm (41). When parental decisions ultimately differ from what providers believe is the right or most appropriate treatment course, either professionally or personally, the uncertainty of the outcome coupled with constraint of parental preference may produce profound moral distress for providers (42). This can be exacerbated by significantly differing beliefs and views on which these decisions are based. If a physician or nurse experiences moral distress as a result of disagreement with parental wishes, communication may become filtered through their own views and values, such that it offers more or less hope for achievement of parental goals than truly exists. This has been described as “ethically indefensible” and amounting to “deception” (43).

Consider a child with a long and arduous clinical course fraught with multiple bouts of severe clinical instability. He has undergone several invasive surgical procedures with refractory hypotension and hypoxia leading to irreversible end-organ damage. There has been no progress for months. Despite frequent counseling on the poor prognosis and lack of ability to wean ventilator support, parents continue to advocate for interventions with the goal of extending his life. They request that the medical team minimize discussions around redirection of care as they have expressed their wishes for their child. Physicians rounding on the child find themselves at a loss for contributions to his care plan that will lead to improvement in his clinical status and bedside nurses feel increasingly distressed by the invasive care they must provide. For months, he remains on the ventilator with no evidence of interacting with his surroundings in a meaningful or purposeful manner. When parents are available, they participate in his care. Otherwise, the nursing staff are his primary care-takers. He develops an infection and acutely decompensates. Despite interventions to treat the infection, he ultimately dies. Staff struggled with continuing to provide invasive medical care. Initially there was great distress over the perception that he was suffering. After discussions with the ethics committee and a better understanding of the degree of his neurological injury and appropriate administration of medications, most providers believed inventions were prolonging a short life in the hospital but no longer felt he was suffering. Ultimately, some reported less moral distress after understanding that he was not suffering, that parents needed more time with him, and that his parents needed to see him die under those circumstances to feel that they advocated for him and gave him every chance to survive.

### Periviability

Periviable deliveries and resuscitation remain a controversial topic. Different institutions have various thresholds for resuscitation based on patient-specific factors, local data and outcomes, physician attitudes and maternal characteristics (44). Population-based estimation of outcomes lacks the precision necessary to make decisions on an individual level. The extreme uncertainty and lack of clear evidence or definitive clinical guidelines on periviable resuscitation can make counseling parents and caregivers challenging. In addition, knowledge and experience of the medical team members are recognized as factors that influence providers' personal views and greatly impact thresholds for resuscitation, estimation of mortality risk, and assessment of long-term outcomes they convey to parents (6).

Discussions around resuscitation often occur at a time with high emotions and stress where outcomes are uncertain and decisions are often made quickly. Providers are tasked with guiding parents through the recognition of their own values while providing available evidence to aid in a shared decision-making approach to care. However, the decisions that are ultimately made can remain a moral and ethical challenge for providers when their personal views and values are in opposition to the care plan they are developing (45). The vast majority of the time, neonatologists and parents are able to come to a mutually agreed upon course of action through discussion and goal sharing. In fact, in a cross-sectional study surveying neonatologists about their preferences surrounding periviable resuscitation, physicians chose options that aligned with parental wishes 66% percent of the time, as compared with options supported by institutional guidelines just 34% of the time (46). The agreeable nature of acting in accordance with parental wishes supports previous findings that understanding parental values and aiding in developing a care plan based on those values results in a lower frequency of moral distress. Further, defaulting to parental wishes and realizing there is no clear right or wrong option despite variation in personal views could partially remove the burden of decision-making from the neonatologist and reduce the moral dilemma he or she would otherwise face. There may also be an element of constraint from institutional guidelines that parental preference helps to override. Rather than strict criteria, guidelines may incorporate parental discretion and offer options that can be tailored to a particular patient. In this way, uncertainty is acknowledged, and shared decision-making can proceed with less impact from medical team bias or distress. A better understanding of the complexities involved in prognostication and decision-making can foster discussions and address discomfort or questions individuals may have (45).

### Conflict and Disagreement

While differing views and opinions can result in discussions that encourage progress and novel ideas, it can also serve as a nidus for moral distress and residue that degrades the relationship between members of the care team and with the family (13, 47). Differences in the application of the zone of parental discretion may lend itself to variability in the amount of parental latitude given in determining interventions and treatment goals. Physicians may be viewed as overindulgent to seemingly unreasonable requests or too inflexible in incorporating parental perspectives. Physicians may agree to parents' wishes if those wishes do not constitute medical neglect while nurses feel distressed at having to carry out interventions that they feel cause more harm than good (48). Consultants may struggle to find helpful recommendations for a case they feel is futile. Different teams caring for the same patient may have opposing recommendations that can erode confidence and trust of the family and care team (13, 47, 49). In these situations, achievement of consensus despite initial disagreement may ultimately allow parents and caregivers to justify their decisions internally and serve to assuage discomfort associated with such ethical dilemmas.

## Addressing Moral Distress

As moral distress can have a significant impact on providers, patients, their families, and the care management plans that are derived, it is imperative that providers go beyond recognizing moral distress and develop effective ways to address it (50). This can lead to improved moral resilience, the capacity to tolerate moral distress and effectively function while mitigating the negative effects (49). While improving moral resilience is greatly dependent upon an individual's internal resources and ability to navigate ethically challenging situations, mindful and intentional reflection of the situation is pertinent to help providers process their emotions. There needs to be training around emotional support and coping for healthcare providers (18).

One of the most important initial steps is recognizing moral subjectivity. This can promote open discussions about the different views and perspectives of others that are driving their opinions and lead to a feeling of comradery. As a result, a shared sense of burden and responsibility for the decisions that are made can develop (5). Specific to end-of-life settings, studies suggest that higher levels of emotional intelligence may temper the negative effects of moral distress (20). By being able to recognize and reflect on emotions and incorporate them with cognitive reasoning, one can more productively manage and cope with morally challenging situations rather than having them lead to moral distress. Ultimately, this leads to improved interactions with patients and decreased levels of stress and anxiety (20). An important factor in this practice is improved and effective communication skills amongst all team members.

There have been many suggestions to support recognizing and addressing moral distress. Included in these suggestions are workshops, debriefings, ethics training, and practices such as reflective writing (20, 51, 52). These methods all have a uniting theme of bringing to the forefront the emotional impact of various situations and events. Writing, specifically, tasks an individual with critically examining the experience as a way of processing and coping. Narrative medicine can aid with coping and enhance physician empathy (18). While studies in other avenues have shown a positive effect in this type of interventions, a small study for NICU nurses in Iran failed to show a difference in moral distress intensity or frequency after 8 weeks of narrative writing (53). This may suggest that there are more complex factors than the act of writing about traumatic events that must occur in order for the practice to be effective. Beyond these measures of personal reflections, the way in which an individual approachs conversations and arrives at or navigates through challenging decisions can have a positive impact on moral distress.

### Counseling and Decision-Making

Since the early 2000's, decision-making in neonatology has seen a shift in practice from providing information to parents and recommending a course of treatment based on evidence alone to a shared decision-making process. In this collaborative approach, factors such as parental values, emotions, trust in the care being provided, life experiences, goals of care, and other personal considerations are taken in to account when developing a care plan (54). The medical team and family must balance the often competing principles of autonomy, beneficence, and non-maleficence. This model of care allows for various stakeholders to express their personal views and values that help them determine a care plan. Values that predominate among healthcare professionals are intertwined with their own personal values. They often emphasize concepts such as patient dignity, quality of care, integrity, a duty to “do no harm,” and alleviate suffering. There are times when their personal views of quality of life vastly differ from that of the parents. As a result, while a shared decision-making approach ensures that all members invested in the care of the baby have input, there are still situations where providers enact a treatment plan they do not feel is optimal for their patient (5). The highest level of distress reported by nurses occurs when following family's wishes when the medical team perceived they were doing “too much” (55). Regardless of the providers' personal views, through the processes of shared decisions-making, he can better understand the views and values of the family. Through recognizing important factors for them in determining quality of life or an appropriate care plan, the provider can make recommendations that align with those views rather than their own personal views. This understanding and approach to navigating goals of care or treatment plans can lead to confidence that the care plan established is a collaborative approach to care that is most appropriate for the individual baby and family regardless of the providers' personal views. As a result, this approach to counseling and decision-making can lead to decreased moral distress.

### Continuity of Care

Inherent in successful shared decision-making is the cultivation of a relationship between the neonatologist, other members of the medical team, the patient, and their parents or other surrogate decision makers. Due to the nature of prematurity and critical illness in newborns, it is not uncommon for a child and their family to encounter multiple care teams throughout their NICU stay, with physician teams rotating approximately every 1–4 weeks. While nursing care teams are typically more consistent, they also involve some degree of variability. Though the same team members may come and go throughout the hospitalization, they participate in different parts of a given patient's care. This can make it difficult for parents to develop consistent relationships that foster trust and an understanding of values.

Despite efforts at standardization and adherence to evidence-based guidelines, there are often differences in the care provided due to the relative paucity of definitive data and algorithms for treatment in neonates. Constraint distress may be experienced due to a plan set in place by another physician or care team and in turn, bedside nurses or other staff members may be faced with the dilemma of aligning with the changing recommendations of the medical team while advocating for frustrated parents and original care plans (5, 16). Their personal views on the best course of action may also make counseling families or communicating with other staff more difficult, thus impacting overall the team's decision-making ability.

Watching a patient suffer due to poor communication and lack of continuity of care leads to high levels of self-reported moral distress (14, 23, 49). It is important with the multiple hand-offs and transitions in care that there are detailed conversations between care providers about the conversations that have taken place and that the unit has a collaborative approach to care. This will allow the providers to better understand how families make decisions, what information has been conveyed to them, and what the expectations for the care plan and treatment options are. Standardizing this approach can lead to seamless care despite changing providers. All providers should be engaged in methods to promote teamwork and to work collaboratively. It is important to ensure all members of the multidisciplinary team have an opportunity to share their thoughts and information to truly promote an environment of shared understanding and community (8). There should be clear methods in place to address conflict or disagreements that may arise among team members (12). It will also allow the family to feel more confident that the providers know their child and decreases the chances of abrupt changes in the care plan.

### Education and Communication

An integrative review examining moral distress in NICU nurses around palliative care found that experiences of moral distress were variable (56). Factors associated with a higher level of moral distress included conflicts among care providers, lack of continuity of care, perceived futile care, false hope, and fluctuation in patients' clinical status. In response to recognizing moral distress, many centers have developed formal and informal educational opportunities to attempt to address causes of distress (18). While the etiology of moral distress appear multifactorial, certain interventions appear to alleviate moral distress. Education around end-of-life care, formation of a care team with the focus of establishing goals of care, and a protocol with a clear plan to address the dying process tend to decrease moral distress.

One center developed comprehensive educational interventions with modules to address nursing moral distress around end-of-life care (57). Overall comfort level with dying patients increased after participating in the educational program. After the educational sessions, nurses reported less compassion fatigue and noted that learning about self-care was important. They also benefited significantly from educations around legal and ethical issues surrounding neonatal end-of-life care. The importance of communication and the many emotions surrounding these situations was also highlighted. Through educational sessions, the importance of the desire for support was recognized. Regardless of the format, having avenues to discuss situations and allow staff to feel supported and heard is critical. Providing support through shared experience is beneficial and a way to foster meaningful relationships (18).

Specific training around communication and environments conducive to meetings and support for staff beyond traditional debriefings is crucial (12). It is known that that those who are less informed about outcomes may make a moral judgement based on misinformation or lack of information and experience moral distress as a result of treatment decisions that challenge their judgment (6). Clear and consistent communication with all members of the team regarding rationale, expected outcomes and options provided to the parents may help to alleviate distress that occurs as a result of simply “not knowing.” To facilitate communications in an educational environment, some centers have introduced rounds or designated sessions for multidisciplinary teams to discuss challenging patients. One center described their approach to a multidisciplinary conference with the goal of promoting communication and consensus building (58). In a structured setting, all members of the NICU staff were invited to attend a discussion about a patient where there was distress or concerns related to the patient care. Input is sought from all team members as well as invited ethicists with the hope of navigating through the complex issues with the goal of improving communication and collaboration to reach a consensus about the case at hand. It appeared that participants felt these conferences took place later than they should in the course of care. Comfort with expressing distress directly correlated to the perceived support of the institution. While communication was cited as a cause of distress, the sessions were attended predominately by those who already felt comfortable recognizing and addressing distress. As a result it was suggested that with education around communication there may be an improved comfort level with crucial conversations that would lead to improved comfort in attending such sessions with the intent of improving patient care and decreasing moral distress.

### Role of Ethics Consultation

With the frequency of ethically challenging situations in the NICU, the utilization of ethics consultations is common. These consultations can serve as forum to promote discussions on moral subjectivity and clarifying ethical challenges that arise (5, 59). This can aid in supporting moral resilience and alleviating compassion fatigue. Recommendations may vary from one institution to the other and laws vary from state to state. Lack of futility clauses and vague language regarding definitions of benefit and harm further complicate many complex clinical situations. In most cases, physicians and nurses feel the ethics committee role is to give advice and help promote a better understanding of the situation. In cases where parents and physicians disagree despite open communication an education, team members feel the ethics committee should make the decision on the most appropriate care plan. A small minority of providers would allow courts and the legal system to make the final decision in the case of clear disagreement (60).

Without clear disagreement, despite moral dilemma physicians and nurses would rather be involved with making the decisions. An ethics committee can help facilitate thoughtful discussions and open communication to better understand the issues that are leading to distress. Through these discussions, the important distinction between an ethical dilemma and moral distress can be made. There can be multiple treatment options that are acceptable while personal views on the most appropriate path may differ. By allowing thoughtful discussions about these options and why they are ethically acceptable, providers may have less moral distress as they have a better understanding in the complexity behind such decisions and also have an opportunity to express their views and concerns (49). Beyond that, ethics committees can help the team navigate communication with parents while understanding that the parents have ultimate responsibility of acting as moral agents for their baby (61). It is only with the recognition of the moral and ethical dilemmas unique to the NICU that organizational support for establishing ethical framework to support complex decision-making can truly come to fruition (62). With this support and structure in place, there can be improved quality of care through less conflict and distress amongst team members.

## Discussion

Moral distress is far more prevalent than even the current literature describes. It transcends healthcare professions and affects the entire healthcare team, the family, and most importantly the patient. While historically described as a negative emotion resulting from poor communication, discrepant values, and paternalistic hierarchy, it can be a source of growth and progress if leveraged correctly. However, if left unaddressed, it can contribute to burnout, job dissatisfaction and, ultimately, a lower quality of care provided.

The multitude of circumstances that can lead to moral distress require a thoughtful and tailored approach to patient care. Ethics rounds, debriefing after deaths, codes, or other challenging situations, and exceptional communication among team members and with caregivers are the cornerstones of minimizing the negative impact of moral distress while leveraging its role in progress. Understanding that different team members will feel moral distress to varying degrees is imperative. Offering options, such as the choice to abstain from care with which they disagree, may be one way to combat constraint in those who are most at risk to feel powerless or “without a voice.” Simulation or case-based discussion can provoke thought and acknowledgment of one's own feelings in various situations so that providers feel confident in their approach to care. In addition, not delaying difficult conversations with parents can minimize the trauma associated with aggressive medical interventions or end-of-life care and will encourage open, deliberate communication. This will allow providers and families to feel that they made the most informed decision.

Most importantly, perhaps, is the recognition of moral distress as an entity, its impact on care provided, and helping staff identify its presence. By acknowledging the influence and implications of moral distress, providers are better equipped to minimize the negative effects and provide safer, more resilient care. Utilization of behavioral medicine resources, team discussions, mentorship and buddy systems for emotional and moral support can encourage self-awareness and a focus on addressing moral distress. Fostering a culture of openness, ethical sincerity and support for those who are struggling can reduce consequences such as burnout and job dissatisfaction. Communication with team members can offer insight in to other perspectives and guide future actions. Guidance from seasoned mentors and reassurance from colleagues regarding the normalcy of moral distress and advice for managing it provides the tools for personal and professional growth.

The impact of moral distress on decisions made in the NICU is under-recognized and represents a potential area for improvement in communication among staff members and with parents. Future areas of investigation should focus on the zone of parental discretion and its boundaries. In addition, given that physicians are most often involved in the medical decision-making for a child, further research on optimal interventions for moral distress tailored to address the specific challenges of different professions would aid in providing additional tools to combat moral distress.

## Author Contributions

MM conceptualized and drafted the initial manuscript. DC aided in conceptualizing, designing, and editing the manuscript. All authors approved the final manuscript as submitted.

## Conflict of Interest

The authors declare that the research was conducted in the absence of any commercial or financial relationships that could be construed as a potential conflict of interest.

## Abbreviations

NICU: Neonatal Intensive Care Unit.
